# Empirical antifungal therapy for health care-associated intra-abdominal infection: a retrospective, multicentre and comparative study

**DOI:** 10.1186/s13613-024-01333-y

**Published:** 2024-06-25

**Authors:** Djamel Mokart, Mehdi Boutaba, Luca Servan, Benjamin Bertrand, Olivier Baldesi, Laurent Lefebvre, Frédéric Gonzalez, Magali Bisbal, Bruno Pastene, Gary Duclos, Marion Faucher, Laurent Zieleskiewicz, Laurent Chow-Chine, Antoine Sannini, Jean Marie Boher, Romain Ronflé, Marc Leone

**Affiliations:** 1 Department of Anesthesiology and Intensive Care Unit, Institut Paoli-Calmettes, Marseille, France; 2 Department of Anesthesiology and Intensive Care Unit, Nord Hospital, Assistance Publique Hôpitaux Universitaires De Marseille, Aix Marseille University, Marseille, France; 3 Réanimation et Surveillance Continue Médico-Chirurgicales Polyvalentes, Centre Hospitalier du Pays d’Aix, Marseille, Aix-en-Provence, France; 4 Biostatistics and Methodology Unit, Institut Paoli-Calmettes, Marseille, France; 5 Aix Marseille University, INSERM, IRD, SESSTIM, Marseille, France

**Keywords:** Intensive care, Health care-associated intra-abdominal infection, Candidiasis, Empirical antifungal therapy, Death rate

## Abstract

**Background:**

Current guidelines recommend using antifungals for selected patients with health care-associated intra-abdominal infection (HC-IAI), but this recommendation is based on a weak evidence. This study aimed to assess the association between early empirical use of antifungals and outcomes in intensive care unit (ICU) adult patients requiring re-intervention after abdominal surgery.

**Methods:**

A retrospective, multicentre cohort study with overlap propensity score weighting was conducted in three ICUs located in three medical institutions in France. Patients treated with early empirical antifungals for HC-IAI after abdominal surgery were compared with controls who did not receive such antifungals. The primary endpoint was the death rate at 90 days, and the secondary endpoints were the death rate at 1 year and composite criteria evaluated at 30 days following the HC-IAI diagnosis, including the need for re-intervention, inappropriate antimicrobial therapy and death, whichever occurred first.

**Results:**

At 90 days, the death rate was significantly decreased in the patients treated with empirical antifungals compared with the control group (11.4% and 20.7%, respectively, p = 0.02). No differences were reported for the secondary outcomes.

**Conclusion:**

The use of early empirical antifungal therapy was associated with a decreased death rate at 90 days, with no effect on the death rate at 1 year, the death rate at 30 days, the rate of re-intervention, the need for drainage, and empirical antibiotic and antifungal therapy failure at 30 days.

**Supplementary Information:**

The online version contains supplementary material available at 10.1186/s13613-024-01333-y.

## Introduction

Health care-associated intra-abdominal infection (HC-IAI), which is one of the most frequent infections among intensive care unit (ICU) patients, was associated with high morbidity and mortality rates [1–3]. In several observational studies, the presence of *Candida* spp. in the peritoneal fluid was associated with an increased death rate [4–7]. Some limited evidence supports the efficacy of empirical or pre-emptive strategies, particularly in patients who have undergone major abdominal surgery [8, 9]. From these findings, current North American guidelines recommend empirical antifungal therapy (EAF) in patients who experienced recent abdominal surgery without obvious or suspected complications such as perforations or leaks and without any other known cause of fever [10]. With some variance, the French guidelines recommended initiating EAF in severe peritonitis in the presence of at least three of the following four criteria: haemodynamic failure, female gender, supra-mesocolic surgery and antibiotic therapy for more than 48 h [11], grounded in an observational study [6].

A rapid overview of the literature suggests a paucity of data supporting these guidelines. Indeed, a seminal study found an association between the presence of yeasts in the peritoneal fluid and increased mortality, but the administration of antifungal therapy did not improve the outcome [5]. In an exploratory study where patients undergoing emergency gastrointestinal surgery for IAI were randomly assigned to the use of a pre-emptive approach with echinocandin or placebo, no evidence was found in favour of the use of pre-emptive antifungal therapy [12]. In contrast, Jacobs et al. found that compared with isotonic saline use, fluconazole use increased the survival of patients with septic shock, and this effect was significant for the patients with intra-abdominal sepsis [13]. At the bedside, antifungal therapy was initiated in 70% of the patients with risk factors for intra-abdominal candidiasis, with no effect on mortality [14].

An ongoing randomised controlled trial aims to compare caspofungin with placebo for the treatment of ICU patients with intra-abdominal candidiasis (NCT03580733). However, since patients are still being recruited for the study, it seems critical to obtain information on the possible effects of antifungal therapy on ICU patients with HC-IAI. Indeed, if the antifungal therapy is ineffective in these patients, such therapy may be associated with adverse effects [15, 16]. Along this line, antifungal therapy should be included in the antimicrobial stewardship policy, by definition, excluding unnecessary antimicrobial agents [17]. Thus, we conducted a retrospective study hypothesizing that EAF therapy was not associated with decreased mortality of ICU patients with HC-IAI. Our primary endpoint was the death rate at 90 days, our secondary endpoints were the death rate at 1 year, a composite outcome assessed 30 days after the HC-IAI diagnosis including death and major complications. In order to preserve the prescription of antifungal agents, we also sought to identify specific subgroups of high-risk patients likely to benefit from a more restricted EAF therapy.

## Methods

### Study design and participants

A retrospective multicentre study was conducted in three French ICUs located in a cancer centre, a general hospital and an academic institution. We included ICU adult patients who contracted HC-IAI after an initial abdominal surgery from 1 January 2009 to 31 December 2021. This study was approved by the Institutional Review Board of the Paoli Calmettes Institute (FONGI-POP IPC 2023−042), which waived the need for written consent, according to the French legislation. Patients and their relatives were informed of the possibility of the use of their medical data for retrospective studies via the Portail d’Accès aux Données de Santé, Assistance Publique-Hôpitaux de Marseille (PADS22-378) and Mes données de santé (https://mesdonnees.unicancer.fr/).

The inclusion criteria consisted of adult patients (> 18 years) requiring the need for source control for HC-IAI occurring after initial abdominal surgery and ICU admission. These patients were classified into two groups: those receiving EAF therapy within 24 h following the HC-IAI diagnosis (EAF group) and those not receiving this therapy (control group).

The exclusion criteria were patients deprived of liberty, under guardianship, with contraindication to the use of antifungal therapy; death within 24 h of HC-IAI; missing information in electronic medical records; and therapeutic limitations within the first 24 h of ICU admission.

### Definitions

HC-IAI was defined as an infection occurring within 60 days following an interventional procedure or any surgery performed in the peritoneal or retroperitoneal space showing gross pus or purulent effusion in the peritoneal cavity or several collections [18].

Re-intervention, empirical antimicrobial therapy and inappropriate empirical antimicrobial therapy are defined in Supplemental Material 1. Source control was defined as the physical measures used to control an invasive infection site and to restore optimal function to the affected area. Four basic elements were considered as source control: drainage, debridement, device removal and definitive measures (usually surgical measures) [19]. Sepsis was defined as life-threatening organ dysfunction caused by a dysregulated host response to infection. Organ dysfunction can be identified as an acute change in total sequential organ failure assessment (SOFA) score ≥ 2 points consequent to the infection. Septic shock was defined as a sepsis and vasopressor therapy needed to elevate mean arterial pressure ≥ 65 mmg Hg and serum lactate > 2 mmol/L (18 mg/dL) after adequate fluid resuscitation [20].

### Management

In the three ICUs, the patients with HC-IAI were managed in accordance with local protocols based on international and national guidelines. The use of antifungal therapy relied on guidelines recommending their introduction in the patients with severe IAI, mainly in the case of health care-associated infection [11, 20, 21]. As this study reflected real-life practice, a direct examination of yeasts in the intra-abdominal samples was not systematically performed. In the three ICUs, local protocols suggested initiating antifungal therapy in patients with HC-IAI and at least three of these four risk factors: haemodynamic failure, female gender, upper gastrointestinal perforation and antibiotic therapy for more than 48 h [6].

### Data collection

The main variables collected at the time of the initial surgery were age, gender, American Society of Anesthesiologists (ASA) score, Charlson Comorbidity Index, body mass index (BMI), sequential organ failure assessment (SOFA) score, surgical or endoscopic procedure, cancer status, type of cancer, emergency status and risk factors for invasive candidiasis [yeast colonisation (< 3 months), if available, and antifungal use in the previous 3 months] (Table 1).

**Table 1:** Characteristics of patients

	Empirical antifungal group (n = 93)	Control group (n = 178)	p-value
Initial surgery			
Age, years	66.4 (13.8)	63.9 (14.4)	0.081
Female gender	38 (40.9)	78 (43.8)	0.735
Charlson comorbidity index	4.6 (2.6)	4.3 (2.8)	0.144
ASA score			0.188
1	7 (7.6)	11 (6.2)	
2	31 (33.7)	82 (46.3)	
3	47 (51.1)	77 (43.5)	
4	7 (7.6)	7 (4.0)	
Body mass index, kg/m^2^	24.2 (5.9)	24.3 (5.6)	0.615
Malnutrition^a^	24 (25.8)	37 (20.8)	0.432
Metastasis	9 (9.7)	34 (19.1)	0.066
Preoperative chemotherapy	22 (23.7)	49 (27.5)	0.587
Preoperative radiotherapy	8 (8.6)	21 (11.8)	0.548
Long-term steroid therapy	4 (4.3)	2 (1.1)	0.210
Previous antibiotic treatment (3 months)	30 (32.3)	37 (20.8)	0.038
Parenteral nutrition	9 (9.7)	14 (7.9)	0.611
Central venous catheters	31 (33.3)	73 (41)	0.217
SOFA score the day of initial surgery	2.1 (2.7)	1.3 (2.2)	< 0.001
Known yeast colonization (3 months)	10 (10.8)	6 (3.4)	0.030
Previous antifungal treatment (3 months)	8 (8.6)	1 (0.6)	0.002
Emergency procedure	25 (26.9)	16 (9.0)	< 0.001
Endoscopic procedure	8 (8.6)	22 (12.4)	0.464
Cancer surgery	64 (68.8)	141 (79.2)	0.081
Gastrointestinal cancer	30 (32.3)	73 (41.0)	0.201
Pancreatic cancer	14 (15.1)	18 (10.1)	0.318
Gynecological cancer	4 (4.3)	31 (17.4)	0.004
Gastric cancer	6 (6.5)	6 (3.4)	0.390
Others	10 (10.8)	13 (7.3)	0.362
Surgery duration > 5 h	22 (23.7)	64 (36.0)	0.054
Perioperative period			
Delay between initial surgery and HC-IAI, days	14.7 (26.4)	10.1 (27.6)	0.013
Delay to surgery, days^b^	1.0 (4.4)	1.4 (4.4)	0.316
Antipseudomonal antibiotics use	86 (92.5)	151 (84.8)	0.083
Aminoglycoside antibiotic use	53 (57.0)	72 (40.4)	0.011
HC-IAI source control			
CT scan	80 (86.0)	146 (83.0)	0.633
Surgery procedure	93 (100)	176 (99)	0.548
Percutaneous or endoscopic procedure	0 (0)	2 (1)	0.755
Causes of HC-IAI			
Upper gastrointestinal perforation	60 (64.5)	63 (35.4)	< 0.001
Colon perforation	9 (9.7)	35 (19.7)	0.052
Small bowel perforation	18 (19.6)	30 (16.9)	0.618
Anastomotic leakage	52 (55.9)	82 (46.1)	0.158
Others	16 (17.2)	30 (16.9)	1
Risk factors > 2^c^ (yeast in peritoneal fluid)	51 (54.8)	27 (15.2)	< 0.001
Empirical echinocandin use	53 (57)	0	< 0.001
Empirical fluconazole use	40 (43.0)	0	< 0.001
SOFA score the day of reoperation for HC-IAI	6.8 (3.4)	4.4 (3.1)	< 0.001
Delta SOFA^d^	4.92 (4.2)	3.1 (2.8)	< 0.001
SAPS II	49.7 (17.8)	36.6 (16.6)	< 0.001
Blood transfusion	42 (45.2)	31 (17.4)	< 0.001
Invasive mechanical ventilation	74 (79.6)	72 (40.4)	< 0.001
Renal replacement therapy	23 (24.7)	13 (7.3)	< 0.001
Vasopressors	75 (80.6)	79 (44.4)	< 0.001
Treatment limitations^e^	1 (1.1)	3 (1.7)	1
Management			
Inappropriate empirical antibiotic therapy including^f^	29 (31.2)	56 (31.5)	1
Inactivity against the pathogen^g^	14 (15.1)	31 (17.4)	0.800
Need for incrementing the antibiotic spectrum for clinical worsening	5 (5.4)	6 (3.4)	0.519
Need for subsequent use of antifungal therapy	-	12 (6.7)	–
Reintervention	31 (33.3)	28 (15.7)	0.001
Percutaneous drainage (CT-scan)	8 (8.6)	13 (7.3)	0.704
Surgery	23 (24.7)	16 (9)	0.001
Death at 30 days	12 (12.9)	16 (9.0)	0.427

Results are expressed as mean (standard deviation) or n (%)

ASA score, American Society of Anesthesiologists Physical Status Classification System; SOFA, Sequential Organ Failure Assessment; HC-IAI, health care-associated intra-abdominal infection; CT, computed tomography; SAPS II, Simplified Acute Physiology Score II

^a^Body mass index < 18.5 or weight loss of over 3 kg in 3 months

Delay between HC-IAI diagnosis and re-intervention

^b^Risk factors: hemodynamic failure, female gender, upper gastrointestinal perforation, and antibiotic therapy for more than 48 h

^c^Difference between the SOFA score on the day of the initial surgery and the SOFA score on the day of the reoperation

^d^During ICU stay

^e^All patients received antibiotic therapy

^f^Based on in vitro susceptibility testing

The main variables collected at the time of re-intervention for HC-IAI were location of the complication, use of vasopressors, SOFA score, mechanical ventilation and renal replacement therapy. For antimicrobial therapy, we collected data regarding the appropriateness of empirical antimicrobial therapy within the first 24 h after the HC-IAI diagnosis.

### Microbiological documentation

In the setting of HC-IAI, blood and peritoneal fluid samples were collected and cultured in accordance with international standards [22]. If available, microbiological colonisation data were collected within the three months preceding the initial surgery.

### Outcomes

The primary endpoint was the death rate at 90 days. The secondary endpoints were composite criteria evaluated at 30 days following the HC-IAI diagnosis, which included the need for re-intervention, inappropriate antimicrobial therapy and death. In addition, we collected the death rate at one year and also sought to identify specific subgroups of high-risk patients likely to benefit from a more restricted EAF therapy.

### Statistical analysis

All of the data are presented as rates (percentages) for the qualitative variables and as means and standard deviations (SDs) for the quantitative variables. The data about the two groups of patients—EAF group versus control group—were compared using the Mann‐Whitney test for continuous variables and the chi‐square or Fisher’s exact tests for categorical variables. All p values < 0.05 were considered statistically significant. We performed a logistic regression analysis to identify the independent determinants associated with the use of EAF therapy as measured by the estimated odds ratio (OR) and 95% confidence interval (CI). Factors with significance or borderline significance (p < 0.1) in the univariate analyses and those identified as pertinent factors in the literature were then included in a multivariable regression model with backward stepwise variable selection. We chose 0.1 as the critical p value for entry into the model and > 0.1 as the p value for removal. The required significance level was set at a p value < 0.05. To further explore the influence of EAF therapy on the death rate at 90 days and the occurrence of the composite criteria at 30 days, we performed an overlap propensity score weighting analysis, in which each patient’s weight determined one’s probability of being assigned to the opposite treatment group [23, 24]. This model has shown high stability and the ability to obtain precise adjustment in various situations, emphasizing the proportion of the population where the most treatment equipoise exists. The propensity score was computed using logistic regression according to the variables associated with the use of EAF therapy. The covariates for the use of the EAF therapy model included independent factors associated with EAF therapy treatment and relevant clinical variables. The data were expressed as the mean or the proportion for each group; absolute standardised mean differences (SMDs) were used to compare the balance in the baseline covariates between the two groups. The absolute SMD is the absolute value of the difference in the mean between the EAF and the control groups divided by the SD. An absolute SMD≤0.10 indicates good balance. In addition, to account for a possible center effect on weight calculation, we added the center variable as fixed and random effects in the propensity score models used for overlap weighting. The overlap propensity score is described in Supplemental Material 2. All tests were two‐sided, and *p* values < 0.05 were considered statistically significant. The process of identifying clusters in our study involved four main steps, which are detailed in Supplemental Material 3 and Supplementary Fig. 3. First, we considered 66 different clinical and biological characteristics from 271 patients (see Supplemental Material 4). Second, we used a factor analysis of mixed data (FAMD) as the dimensionality reduction method given that all 271 patients were initially distributed in a 66-dimensional space. FAMD helped to reduce the 66 dimensions down to a smaller number, capturing 80% of the total variance of the dataset. Thus, the selected dimensions were retained for unsupervised ascendant hierarchical cluster analysis performed in a third step. Outcome and antifungal treatment were therefore not included in this unsupervised analysis for clusters determination. The major clusters were identified according to algorithms and differences among clusters. Comparisons between the clusters were assessed using the Chi-square for qualitative variables and the Kruskal–Wallis test for quantitative variables. A Cox regression model was also used for 90-day survival analysis and adjusted survival curves were used for illustration. Finally, a prediction model construction was performed. We constructed linear support vector machine (SVM) classifier according to the results of the unsupervised clustering. Only variables available at HC-AI diagnosis and at ICU admission (Supplemental Material 4) were randomly divided into 70% training and 30% test dataset. The optimal parameters were found by using the grid search based on tenfold cross validation on 70% training dataset, and the classification performance of the model were tested by testing 30% dataset. Performance values of the classifier were described. Statistical analyses were performed with R statistical software, version 3.4.3 (available at https://www.rproject.org/).

## Results

### Patient description

**Fig. 1:**
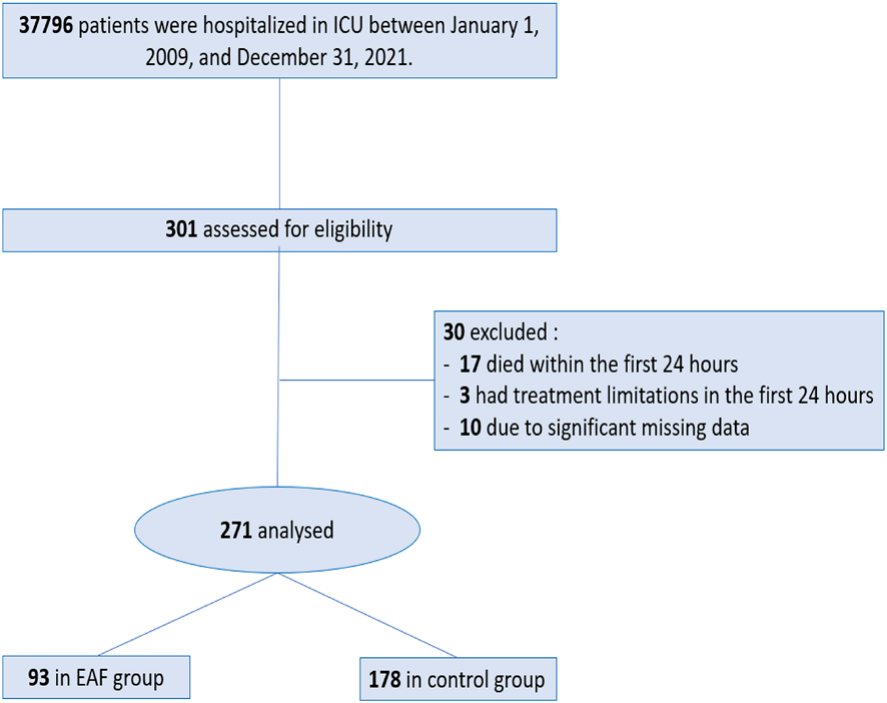
Flowchart of inclusion

During the study period, 301 ICU patients with HC-IAI were eligible for inclusion in the study; of these, 30 patients were secondarily excluded (Fig. 1). Thus, 271 patients were finally included.

The initial surgery was cancer surgery of 64 (68.8%) patients in the EAF group and 141 (79.2%) in the control group (p = 0.81). The initial surgery was an emergency procedure in 25 (26.9%) cases in the EAF group and in 16 (9%) cases in the control group (p < 0.001).

### Source control

The intervals between HC-IAI diagnosis and re-intervention were 1 day (SD = 4.4) in the EAF group and 1.4 days (SD = 4.4) in the control group (p = 0.32). Upper gastrointestinal perforation was reported in 60 (64.5%) and 63 (35.4%) patients in the EAF and the control groups, respectively (p < 0.001). At least three out of the four risk factors were found in 51 (54.8%) patients in the EAF group and 27 (15.2%) patients in the control group (p < 0.001). Source control was performed through surgical procedures in 93 (100%) and 176 (99%) patients in the EAF and the control groups, respectively (p = 0.55).

### Antibiotic therapy

All patients received empirical antibiotic therapy in the first 24 h after the HC-IAI diagnosis. The rates of in vitro inactivity against the identified pathogen were similar in the EAF and the control groups (15.1% versus 17.4%, p = 0.80). Antipseudomonal antibiotics were used in 86 (82.5%) and 151 (84.8%) patients in the EAF and the control groups, respectively (p = 0.08). Aminoglycoside was used in combination with beta-lactam in 53 (57%) patients in the EAF group and 72 (40.4%) patients in the control group (p = 0.01).

### Antifungal therapy

Ninety-three (34%) patients received EAF therapy, while 178 (66%) patients did not receive it (Table 1). Among the 93 patients who underwent EAF therapy, 53 (57%) were treated with echinocandin, while 40 (43%) were treated with azole for 11 days (SD = 10.2). In the control group, 12 (7%) patients received delayed antifungal treatment, due to secondary clinical worsening or after microbiological identification.

### Day of re-intervention

The patients in the EAF and the control groups had SOFA scores of 7 (SD = 3) and 4 (SD = 3), respectively (p < 0.001). All patients in the EAF group received an appropriate antifungal therapy (Table 2). Vasopressors were used in 75 (80.6%) patients in the EAF group versus 79 (44.4%) patients in the control group (p < 0.001). Yeasts were identified in intraoperative peritoneal fluid samples from 29 (31.2%) and 18 (10.1%) patients in the EAF and the control groups, respectively (p < 0.001).

**Table 2:** Microbiological results expressed in number and percentage of infected patients according to the site.

Microbiological variables	All analyzed patients (n = 271)
*Bacterial documentation*^*a*^	
Intraoperative peritoneal fluid samples of HC-IAI	
Positive peritoneal fluid	187 (69.0)
Gram negative bacteria	153 (56.5)
*E. coli*	100 (36.9)
*Klebsiella* spp*.*	33 (12.2)
Nonfermenting bacilli	22 (8.1)
Gram positive cocci	69 (25.5)
*Enterococcus*	56 (20.7)
*Staphylococcus*	8 (3.0)
*Streptococcus*	13 (4.8)
Anaerobes	11 (4.1)
Multidrug resistant bacteria^b^	46 (17.0)
*Blood cultures of HC-IAI*	
Positive blood cultures	64 (23.6)
Gram negative bacteria	59 (21.8)
*E. coli*	23 (8.5)
*Klebsiella* spp	11 (4.1)
Nonfermenting bacilli	9 (3.3)
Gram positive cocci	20 (7.4)
*Enterococcus*	12 (4.4)
*Staphylococcus*	6 (2.2)
*Streptococcus*	4 (1.5)
Multidrug resistant bacteriab	9 (3.3)
Anaerobes	18 (6.6)
*Drain samples collected < 24 h after HC-IAI reoperation*	
Positive drain culture	17 (6.3)
Gram negative bacteria	12 (4.4)
*E. coli*	0
*Klebsiella* spp	5 (1.8)
Nonfermenting bacilli	7 (2.6)
Gram positive cocci	0
Enterococcus	0
*Staphylococcus*	2 (0.7)
*Streptococcus*	1 (0.4)
Multidrug resistant bacteriab	5 (1.8)
Anaerobes	0
*Yeast documentation*	
Yeast at the time of first reoperation	53 (19.6)
Peritoneal fluid	47 (17.3)
*Candida albicans*	33 (12.1)
*Candida glabrata*	10 (3.7)
*Candida tropicalis*	5 (1.8)
*Candida kefir*	0
Blood cultures	10 (3.7)
*Candida albicans*	6 (2.2)
*Candida glabrata*	2 (0.7)
*Candida tropicalis*	1 (0.4)
*Candida kefir*	1 (0.4)
Drain	3 (1.1)
*Candida albicans*	3 (1.1)
*Candida glabrata*	0
*Candida tropicalis*	0
*Candida kefir*	0

Results are expressed as n (%)

^a^Main bacterial documentation

^b^Defined as described by Magiorakos et al.

### Outcomes

#### Naive analysis

The death rates at 90 days were 16.1% (n = 15) and 13.5% (n = 24) in the EAF and the control groups, respectively (p = 0.68). The death rates at one year were 29% (n = 27) and 22.5% (n = 40) in the EAF and the control groups, respectively (p = 0.30). The incidence rates of the composite criteria at 30 days were 48.4% (n = 45) and 37.6% (n = 67) in the EAF and the control groups, respectively (p = 0.12) (Table 3).

**Table 3:** Overlap propensity score-weighted characteristics and clinical outcomes in patients in the empirical antifungal therapy group and the control group.

	Unweighted analysis			Overlap propensity score-weighted analysis		
	Empirical antifungal group (n = 93)	Control group (n = 178)	SMD	Empirical antifungal group	Control group	SMD
Age	66.4	63.9	0.181	66	66	< 0.001
Female gender	40.9%	43.8%	0.071	44.4%	44.4%	< 0.001
Charlson comorbidity index	4.6	4.3	0.132	4	5	< 0.001
Cancer surgery	68.8%	79.2%	0.239	71.5%	71.5%	< 0.001
Previous yeast colonization^a^	10.8%	3.4%	0.291	6.2%	6.2%	< 0.001
Previous antibiotic treatment (3 months)	28.8%	32.3%	0.262	29.8%	25%	0.1
SOFA score the day of initial surgery	2.1	1.3	0.318	1	1	< 0.001
Delta SOFA score^b^	4.9	3.1	0.519	4	4	< 0.001
Delay to surgery^c^	1.0	1.4	0.154	1	1	< 0.001
Upper gastrointestinal perforation^d^	64.5%	35.4%	0.609	54.6%	54.6%	< 0.001

*Outcomes*			p-value			p-value
Death at 90 days	16.1%	13.5%	0.684	11.4%	20.7%	
OR (95% CI)					0.492 [0.262—0.925]	0.02
Composite criteria at 30 days	48.4%	37.6%	0.115	47.3%	43.2%	
OR (95% CI)					1.182 [0.687—2.033]	0.53
Death at 1 year	29.0%	22.5%	0.298	23.6%	29.3%	
OR (95% CI)					0.742 [0.395—1.394]	0.34

Data are expressed as mean or proportion for each group SOFA, Sequential Organ Failure Assessment; OR, odd ratio; CI, confidence interval. The absolute standardized mean difference (SMD) is the absolute value of the difference in mean between treatment groups divided by the SD. An absolute mean standardized difference less than or equal to 0.10 indicates good balance

aKnown yeast colonization in the previous 3 months

bDifference between the SOFA score on the day of the reoperation and the SOFA score on the day of the initial surgery

cDelay between health care-associated intra-abdominal infection (HC-IAI) diagnosis and re-intervention in days

dIn cause of HC-IAI

#### Independent factors associated with the use of EAF

As shown in the multivariate analysis, the factors independently associated with the use of EAF were yeast colonisation (p = 0.003), age (p = 0.004), SOFA score on the day of the first surgery (p < 0.001), delta SOFA score (p = 0.014) and Charlson Comorbidity Index (p = 0.05) (Supplementary Fig. 1).

#### Overlap propensity score weighting analysis

**Fig. 2:**
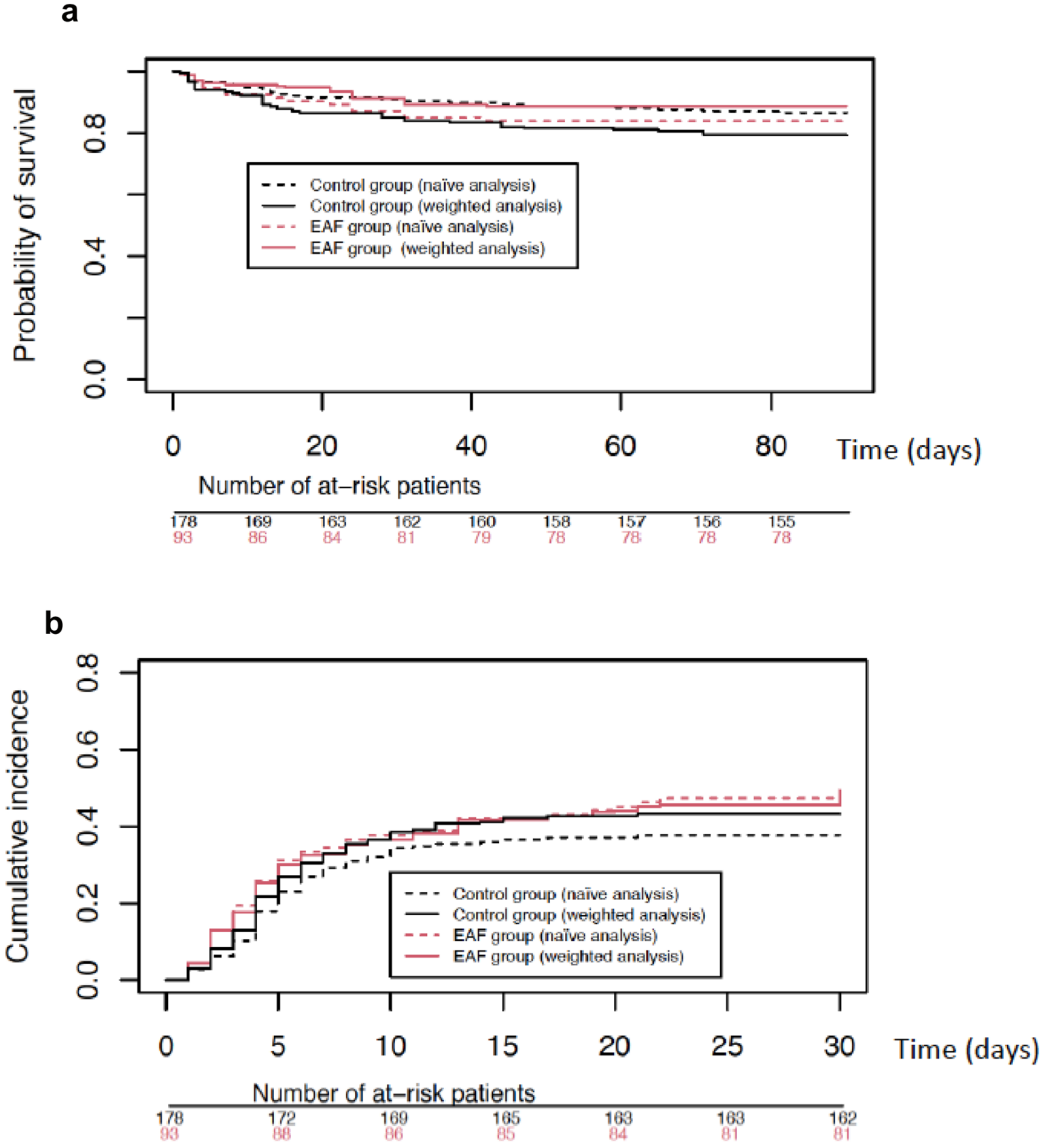
Probability of survival according to empirical antifungal therapy use (naïve and weighted analysis) (**a**), cumulative incidence of the composite criteria occurrence according to empirical antifungal therapy use (naïve and weighted analysis) (**b**). EAF group, empirical antifungal therapy group

We used overlap propensity score weighting to further assess the association between the influence of EAF therapy on the death rate at 90 days and the occurrence of the composite criteria at 30 days. The model was constructed using independent factors associated with the use of EAF therapy and other relevant clinical variables. The final explanatory variables retained were age, sex (female), Charlson Comorbidity Index, SOFA score at initial surgery, variation of SOFA score between initial surgery and the HC-IAI diagnosis, supra-mesocolic surgery, cancer surgery, yeast colonisation at ICU admission, and delay between the first signs of HC-IAI and surgery. After the overlap propensity score weighting, both groups were adequately balanced (Supplementary Fig. 2). The death rates at 90 days were 11.4% and 20.7% (OR = 0.49, 95% CI [0.26–0.92], p = 0.02) in the EAF and the control groups, respectively (Table 3). These results were consistent with the 90-day survival analysis (hazard ratio = 0.22, 95% CI [0.01–0.50], p = 0.044) (Fig. 2a). Considering the center effect as a fixed effect, the death rates at 90 days were 11.2% and 23.2% (OR = 0.42, 95% CI [0.20–0.85], p = 0.016) in the EAF and the control groups, respectively. Considering the center effect as a random effect, the death rates at 90 days were 11.4% and 20.7% (OR = 0.44, 95% CI [0.20–0.95], p = 0.04) in the EAF and the control groups, respectively. The conclusions were unchanged, reinforcing our original model.

At 1 year, 23.6% and 29.3% of the patients did not survive in the EAF and the control groups, respectively (OR = 0.74, 95% CI [0.40–1.39], p = 0.34). The incidence rates of the composite criteria at 30 days did not differ between the two groups (47.3% in the EAF group versus 43.2% in the control group, OR = 1.18, 95% CI [0.69–2.03], p = 0.53) (Fig. 2b).

### Clusters determination

**Fig.3:**
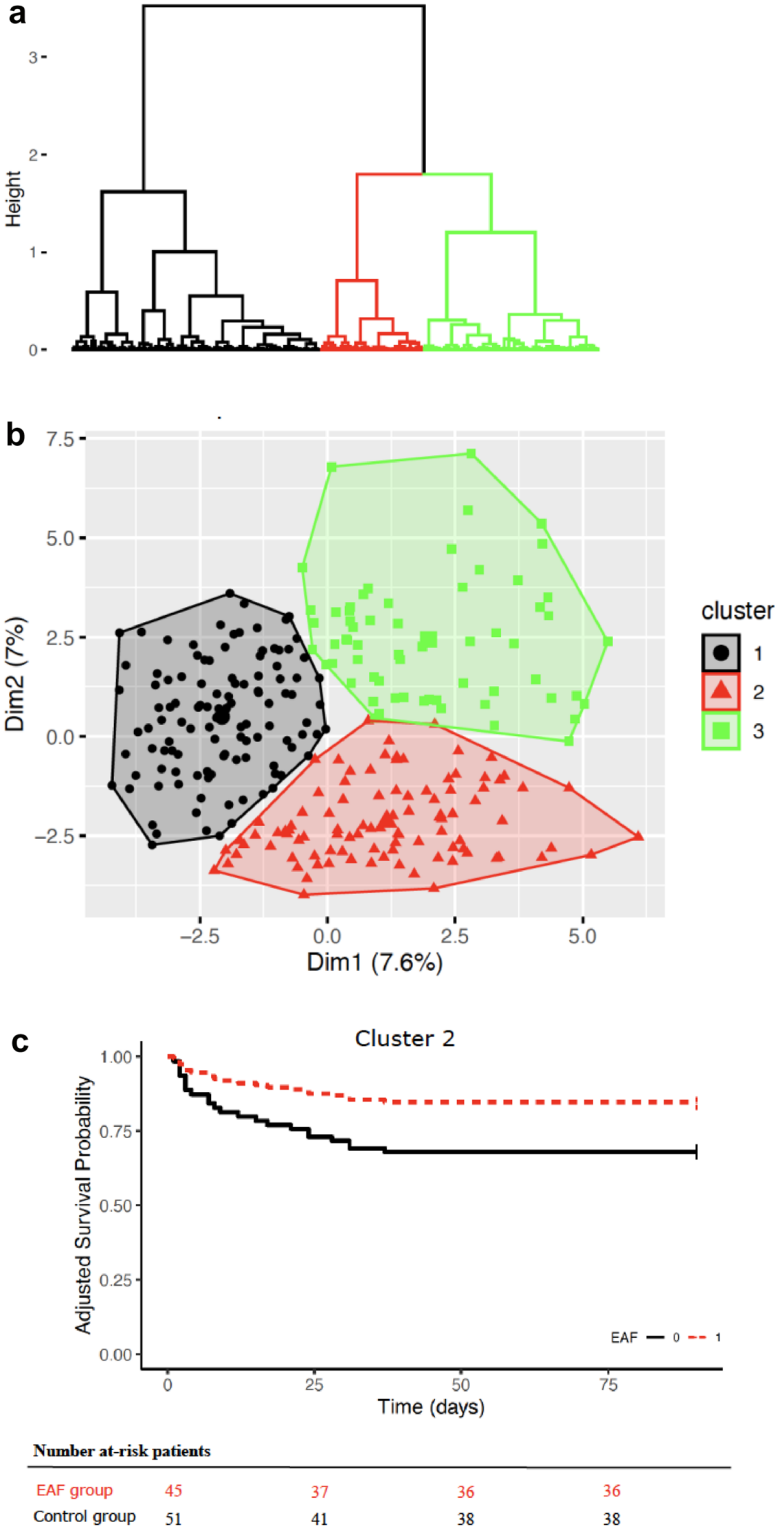
Dendrogram showing the unsupervised ascendant hierarchical cluster analysis forming 3 clusters (**a**), scatterplot showing the 271 patients’ distribution in the 3 clusters in the two first dimensions obtained from the factor analysis of mixed data (FAMD) model (**b**), probability of survival according to empirical antifungal therapy use in the Cluster 2

We performed FAMD on the 66 clinical and biological variables identified by the standard procedure which yielded 66 dimensions. Variances of 66 dimensions decreased gradually, and variances of the top 32 dimensions accounted for more than 80% of the total variance. Thus, the top 32 dimensions were retained for further analysis. A scree plot of these 32 dimensions is shown in Supplementary Fig. 4. The contribution of each variables to the first 5 dimensions are shown in Supplementary Fig. 5. Subsequently, unsupervised ascendant hierarchical cluster analysis was performed with the matrix made with the top 32 dimensions values of 271 patients. Three clusters were identified, the dendrogram showing the ascendant hierarchical cluster analysis forming 3 clusters (Fig. 3a) and the scatterplot showing the distribution of the 271 patients in the 3 clusters are displayed (Fig. 3b). The comparison between the main significant variables of each cluster (Supplemental Material 5) correlated with clusters determination provides insights into cluster definition at the patient’s bedside.

#### Clusters description

- Cluster 1 (low-risk patients, n = 113)

Cluster 1 consisted of a cohort characterized by a low risk, as evidenced by mean SAPS II and SOFA scores of 32 (13) and 3.5 (2.3) respectively after reoperation, both significantly lower than those observed in the other two groups. Within this cluster, a high incidence of cancer was noted (97%, n = 110), contrasting with a complete absence of patients with an ASA score of 4 (n = 0). Initial colon surgery was prevalent (26%, n = 29), whereas supra-mesocolic surgery (11.5%, n = 13) and emergency surgery (0.9%, n = 1) were less frequent. Notably, laparoscopy (43.4%, n = 49) and robotic surgery (9.7%, n = 11) were more commonly performed as compared with other clusters. The majority of patients in this cluster (90%, n = 102) had at least one risk factor for invasive candidiasis, with only 1% of patients (n = 1) meeting more than 2 risk factors for yeast isolation in peritoneal fluid. EAF treatment was administered to 11% of cases (n = 12), and invasive fungal infection (IFI) was diagnosed in only 6.2% of cases (n = 7). The 90-day mortality rate for these patients was 5.3% (n = 6). After adjustment for key confounders (Charlson score and SAPS II score), EAF was not associated with 90-day survival (HR = 2.34, 95% CI [0.26–21.2], p = 0.448), nor was it associated with occurrence of the composite criteria (HR = 1.279, 95% CI [0.446–3.672], p = 0.647).

- Cluster 2 (high-risk patients, n = 96)

Cluster 2 encompassed a population of high-risk patients, as indicated by SAPS II and SOFA scores of 51 (20) and 6.4 (3.8) respectively after reoperation—scores that were notably higher than those observed in other clusters. In this group, cancer was less prevalent (47%, n = 45), but the patients with an ASA score of 4 were more numerous (10%, n = 10). Colonic surgery was frequent (39.6%, n = 38), along with emergency surgery (34.4%, n = 33) and supra-mesocolic surgery (66.7%, n = 64). The majority of surgeries had a duration of less than 5 h (99%, n = 95). While only 27% (n = 26) of these patients presented at least one risk factor for invasive candidiasis, a significant portion—41% (n = 39) had more than 2 risk factors for yeast isolation in peritoneal fluid. EAF treatment was administered in 47% of cases (n = 45), with IFI diagnosed in 19% (n = 18) of cases. For this cluster, the 90-day mortality rate was 22.9% (n = 22). After adjustment for key confounders (Charlson score and SAPS II score), EAF was significantly associated with 90-day survival (HR = 0.391, 95% CI [0.156–0.981], p = 0.045, Fig. 3c), but was not associated with occurrence of the composite criteria (HR = 0.946, 95% CI [0.461–1.291], p = 1.943).

- Cluster 3 (intermediate-risk patients, n = 62)

Cluster 3 comprised an intermediate-risk patient population, as reflected in the SOFA score at 3.15 (2.68) on the day of the first procedure—a score significantly higher than the other two clusters. Moreover, the SAPS II and SOFA scores were 41.69 (14.39) and 6.40 (3.04), respectively, on the day of reoperation. In this cohort, the procedures were most frequently carried out via laparotomy (77.4%, n = 48), often in the context of supra-mesocolic surgery (74.2%, n = 46), particularly involving pancreas (21%, n = 13). The duration of surgery was less than 5 h in half of cases. In this cluster, all patients had at least one criterion for invasive candidiasis, and the majority of them had 2 risk factors for yeast isolation in peritoneal fluid (61.3%, n = 38). EAF treatment was administered in 58% of cases (n = 36), with IFI diagnosed in 45.2% (n = 28) of cases. The 90-day mortality rate for this group was 17.7% (n = 11). After adjustment for key confounders (Charlson score and SAPS II score), EAF was not associated with 90-day survival (HR = 0.47, 95% CI [0.13–1.65], p = 0.239), nor was it associated with occurrence of the composite criteria (HR = 0.946, 95% CI [0.461–1.291], p = 1.943).

### SVM classifier

Using the results of the unsupervised hierarchical clustering, we trained a linear SVM classifier model to aid clinical judgement at ICU admission. Fifty-five clinical and biological predictors available at ICU admission are described in Supplemental Material 4. A grid-search on tenfold cross validation for parameters was performed to find the best model, and parameters producing the best result were chosen (Supplementary Fig. 6). Accuracy was used to select the optimal model using the largest value. On the train dataset, the final value of the cost used for the model was C = 0.01 which corresponds to an accuracy of 0.876 and mean total kappa statistic of 0.807. The confusion matrix for the classifier model on the test dataset is shown in Supplemental Material 6, accuracy was 0.924 and kappa statistic of the model on the test dataset was 0.881, which suggested that the model was not over-fitted. Differences between the trained and the tested models did not reach a significant level (p = 0.26). Positive and negative predictive values for prediction of clusters 1,2 and 3 the first day of HC-IAI in the ICU were 94%, 87%, 100% and 98%, 98% and 94%, respectively (Supplemental Material 7).

## Discussion

Our findings suggest that the use of EAF therapy in ICU patients requiring re-intervention after the initial abdominal surgery was associated with a decreased death rate at 90 days. To our knowledge, only a few studies showed an effect of antifungals on the mortality of these patients [4, 5, 25]. This effect was not sustained in long-term outcomes. The composite criteria measured at 30 days were similar in the two groups.

We found that 51 (65%) out of the 78 patients with risk factors for intra-abdominal candidiasis received EAF therapy [6]. At the bedside, 93 patients received EAF therapy, including 42 (45%) patients who did not have the risk factors [6]. In our study, the magnitude of organ failure at ICU admission, the co-morbidities and a previous colonisation by yeasts were the main independent factors associated with the use of EAF therapy. Our global findings are in line with a previous study that showed moderate adherence to the guidelines for ICU patients [26].

While the presence of *Candida* in the peritoneal fluid was repeatedly associated with increased mortality of patients with HC-IAI, the use of EAF therapy is supported by weak evidence so far. Indeed, in the field of prophylaxis, the use of daily oral fluconazole was associated with a reduced risk of fungal peritonitis in peritoneal dialysis patients [27]. Our results also support those obtained from a study involving 71 patients with septic shock (caused by nosocomial pneumonia or intra-abdominal sepsis), who received either fluconazole or placebo during the septic shock episode [13]. In contrast, in a randomised double-blind multicentre study, the use of a single 400-mg dose of fluconazole during the surgery of patients with intra-abdominal perforations did not affect their death rate, whereas the identification of *Candida* was associated with increased mortality [25].

In our study, a striking finding was the low incidence of *Candida glabrata*, while recent studies showed that *Candida glabrata* could account for 20% of isolates in intraabdominal candidiasis [28]. On the other hand, 43% of our patients who received an empirical treatment were treated by fluconazole. It is therefore important to analyze our results with caution. Indeed, as *Candida glabrata* is known to be resistant to fluconazole, in the case of a high incidence of *Candida glabrata*, our results may have been different.

Our results showed no association between the use of EAF therapy and the rate of abdominal drainage during the first 30 days following the HC-IAI diagnosis. In patients with HC-IAI, it is well-known that the local complications are predominantly due to poor bacterial control [29, 30]. Similarly, in ICU surgical patients with invasive candidiasis receiving prophylactic antifungal therapy, rates of invasive *Candida* infection can be high, with up to 16.5% reported in the literature [31]. Regarding the death rate at 90 days, the mechanisms through which antifungal use may be associated with decreased mortality remain ambiguous. A possible explanation could be the early introduction of EAF therapy, which has been associated with improved survival compared with delayed introduction [32].

As we collected the death rate at 1 year, we did not confirm a sustained significant difference between the 2 groups. At 1 year, the effect of any treatment, even if initially efficient, could be attenuated by the mortality due to the primary reasons for hospitalisation, such as the underlying cancer representing 76% of our patients. Previous studies showed that the death rate of ICU survivors increased by around 20% at 1 year after their ICU discharge [33–35].

In a second step, our study yielded a crucial outcome: the prompt identification, directly at ICU bedside, of three distinct patient clusters associated with varying levels of risk. The clusters 2 and 3 represented the most severe cases while patients in cluster 1 were at low risk and presented a lower prevalence of IFI. Interestingly, EAF therapy was associated to improved survival for Cluster 2 patients. These findings suggest the need for immediate EAF in Clusters 2 and 3, in contrast to Cluster 1. Early identification of high-risk patients, facilitated by an SVM machine learning technique, could judiciously avoid unnecessary use of antifungals in specific cases. While conventional guidelines dismiss the notion of pre-emptive antifungal treatment [10], our study suggests a reconsideration, especially in light of emerging tools such as fungal biomarkers [36]. Additionally, alternative approaches, including machine learning strategies, could play a pivotal role in this context.

We have to acknowledge our study’s limitations. First, due to this study’s retrospective design, undetermined confounding cannot be excluded. However, we conducted a propensity score-based overlap weight analysis, aiming to mimic a randomised controlled trial [23]. The 2 groups shared the same features, making them highly comparable. Second, we did not use a standardized therapeutic approach to HC-IAI. Some guidelines suggested the need for direct examination of peritoneal fluid before introducing antifungals or expecting growth from culture [6, 7, 11]. At the bedside, conducting a direct examination or obtaining the results from culture may be challenging. Hence, the patients received antifungals based on the identified risk factors that were clinically relevant [6]. Third, we did not collect the data on the precise delay of the administration of empirical antimicrobial therapy, but it was systematically administered in the first 24 h after the HC-IAI diagnosis. Fourthly, we do not have details of the surgical procedures, due to their complexity, but we categorized the type of interventions that resulted in source control. Finally, the majority of our study’s participants were cancer patients, and their trajectory at one year may differ from that of non-cancer patients [37].

## Conclusion

Our retrospective, multicentre and comparative study showed an association between the use of EAF therapy and the death rate at 90 days among ICU patients with HC-IAI. This association was not confirmed at one year, and the local evolution was not associated with the use of EAF therapy. This retrospective study reinforces the relevance of ongoing randomised controlled trials in this field.

### Supplementary Information

Supplementary Material 1.

## Data Availability

The datasets used and/or analyzed during the current study are available from the corresponding author on reasonable request.
