# Supplementary material for: Empirical antifungal therapy for health care-associated intra-abdominal infection: a retrospective, multicentre and comparative study
Source: Ann Intensive Care. 2024 Jun 25;14:98. doi: 10.1186/s13613-024-01333-y (PMC11199462; doi:10.1186/s13613-024-01333-y)
Supplement: Supplementary file 1 — Supplementary Material 1. [file 13613_2024_1333_MOESM1_ESM.docx]

**Supplementary Material**

**Table of contents:**

- **Supplementary Material 1 (Definitions)………………………………………page 2**
- **Supplementary Material 2 (Overlap propensity score) ………………………page 2**
- **Supplementary Material 3 (Clusters determination) …………………………page 3**
- **Supplementary Material 4 (FAMD and SVM constructions) ………………. page 4**
- **Supplementary Material 5 (Comparisons between clusters) ………………...page 5**
- **Supplementary Material 6 (Confusion matrix) ……………………………… page 8**
- **Supplementary Material 7 (Statistics by clusters) …………………………... page 8**
- **Supplementary Figure 1 (Multivariate analysis for EAF use) ……………. page 10**
- **Supplementary Figure 2 (Standardized Mean Differences) ………………. page 10**
- **Supplementary Figure 3 (Clustering methodological design) ……………. page 11**
- **Supplementary Figure 4 (Scree plot) …………………………………………page 12**
- **Supplementary Figure 5 (Dimensions characteristics) ……………………. page 13**
- **Supplementary Figure 6 (Accuracy of the SVM classifier) ………………. page 16**
- **References ……………………………………………………………………. Page 17**

1. **Supplementary Material 1**

**Definitions**

- Re-intervention was defined by surgical management of HC-IAI or by non-surgical interventional procedure, which included endoscopic and/or radiologically guided drainage.
- Risk factors for yeast isolation in peritoneal fluid were those described by Dupont^1^: hemodynamic failure (heart rate <54 bpm and/or systolic blood pressure <60 mm Hg and/or ventricular tachycardia or fibrillation and/or arterial pH≤7.27 with PaCO2≤49 mmHg), Upper gastrointestinal perforation, female gender and ongoing antimicrobial therapy≥48H
- Risk factors for invasive candidiasis were those usually described in the recent literature^2^ : immunosuppression, broad spectrum antibiotics >72h, candida colonization, central venous catheter, total parenteral nutrition.
- Empirical antimicrobial therapy was defined as the antibiotic or antifungal treatment initiated within the first 24 hours of HC- IAI diagnosis. Inappropriate empirical antimicrobial therapy was defined by (i) an antimicrobial inactive against the pathogen based on *in vitro* susceptibility testing; (ii) need for incrementing the spectrum of antimicrobial therapy due to clinical worsening; (iii) need for subsequent use of antifungal therapy; and (iv) death within the 30 days following the HC-IAI diagnosis.

1. **Supplementary Material 2**

**Overlap propensity score**

- Overlap propensity score weighted logistic regression models investigated associations between treatment status and the probability of testing positive for 90‐day mortality and occurrence of the composite criteria at 30 days. Subsequently, to validate the robustness of our findings, a weighted Cox regression model was also used for 90-day survival analysis. In both regression models, a bootstrap resampling approach with 1000 samples was applied to enhance the precision of variance estimates and ensure the reliability of our results. Naïve and weighted survival and cumulative incidence curves were used to summarize outcomes.

1. **Supplementary Material 3**

**Clusters determination (Supplementary Figure 3)**

- First, 66 clinical and biological variables from 271 patients (Supplemental Material 4) were included in this analysis. Each variable can be represented as a dimension (or an axis); with the 66 variables selected, the dataset is distributed in a 66-dimensional space. Thus, each patient is represented by a set of values among the 66 variables, creating a point in the 66-dimensional space.
- Second, a factorial analysis of mixed data (FAMD) was performed. We used FAMD as a dimensionality reduction method, given that the 271 patients are initially distributed in a high-dimensional space (n=66)^3^. In practice, pre-processing using the FAMD method (R package FactoMineR) enabled us to deconstruct the principal variances of 66 relevant clinical and biological variables (Supplementary Figure 3) of 271 patients into fewer relevant dimensions (or principal components). FAMD is a combination of principal component analysis for continuous variables and multiple correspondence analysis for categorical variables. All variables were normalized prior to the dimensionality reduction. Missing data were imputed for dimensionality reduction (R package missMDA). Thus, the number of dimensions represented 80% of the total variance were selected and retained for further unsupervised ascendant hierarchical cluster analysis. Outcome and antifungal treatments were therefore not included in this step. Above this level, the cumulative explained variance according to the number of dimensions gradually reaches a plateau (Supplementary Figure 4). The contribution of each variables to the first 5 dimensions are shown in Supplementary Figure 5.
- Third, for clustering, we used hierarchical clustering on principal components (HCPC), (R package FactoMineR). HCPC entails an unsupervised ascendant hierarchical cluster analysis in the first step and k-means clustering to improve the initial clustering. The unsupervised ascendant hierarchical cluster analysis was performed on the dimensions previously retained by the FAMD model, using an ascendant algorithm on the Euclidean distances between points and according to the Ward’s method; it allowed to minimize the total intracluster variance and to generate the dendrogram^4^.Note that the ascendant hierarchical cluster analysis was performed on dimensions following FAMD, and not directly on the original dataset itself. In addition to dimensionality reduction, this additional step is done to reduce noise in the data and generally yields a more stable cluster analysis. We chose a three-cluster solution based on the higher relative loss of the sum of within-cluster variance.^3^ This entails that all participants are empirically divided into three cluster sub-groups. Clusters’ names were defined based on the most frequently clinical and biological situations found in each cluster, in order to summarize and label each group of patients at one glance. Comparisons between the clusters were assessed using the Chi-square for qualitative variables and the Kruskal–Wallis test for quantitative variables*.* A Cox regression model was also used for 90-day survival analysis and adjusted survival curves were used for illustration.
- Finally, we constructed linear support vector machine (SVM) classifier according to the results of the unsupervised clustering. SVM is a supervised learning method that constructs hyperplanes in a high-dimensional space to separate training data into different classes and is often used for classification. An SVM classifier model of clusters was constructed (R package caret). Only 55 variables available at ICU admission (Su) were chosen as the predictor variables, and the response variable was the FAMD-based clustering results. All 271 patients were randomly divided into 70% training and 30% test datasets. We implemented a grid search and 10fold cross validation for tuning and validating the prediction model on the training dataset. Then the model with optimal parameters were tested on the test dataset. Accuracy and Kappa statistic were calculated (R package caret) and used to evaluate the performance of SVM model with different kernel and parameters. Differences between the train and test models were evaluated using Mc Nemar test (significant if p<0.05). Sensitivity, specificity, positive predictive value, negative predictive value, prevalence, detection rate, detection prevalence and balanced accuracy were also provided.

1. **Supplementary Material 4 : Clinical and biological variables used to realize factor analysis of mixed data (FAMD) (n=66) and SVM classifier (n= 55 in bold):**

[**1] Age**

**[2] Female gender**

**[3] ASA score (American Society of Anesthesiologists Physical Status Classification System)**

**[4] BMI (body mass index)**

**[5] Charlson CI (Charlson comorbidity index)**

**[6] Malnutrition**

**[7] Metastasis**

**[8] Cancer**

**[9] Emergency surgery**

**[10] Rectal surgery**

**[11] Colectomy**

**[12] Hepatic surgery**

**[13] Pancreatic surgery**

**[14] Small bowel surgery**

**[15] Gastrectomy**

**[16] Simple digestive continuity restoration**

**[17] Combined surgery with colectomy**

**[18] Total hysterectomy**

**[19] Urologic surgery**

**[20] Endoscopic procedure**

**[21] Other surgery**

**[22] Laparotomy procedure**

**[23] Laparoscopy procedure**

**[24] Robotic surgery**

**[25] Surgery duration>5h**

**[26] Antibiotic as risk factor (ongoing antimicrobial therapy≥48H**)^1^

**[27] Hemodynamic as a risk factor (hemodynamic failure)**^1^

**[28] Supramesocolic surgery"**

**[29] Risk_factors>2 (risk factors for invasive candidiasis)** **^2^**

**[30] At least one risk factor (risk factors for invasive candidiasis)** **^2^**

**[31] Nb of risk factors (risk factors for invasive candidiasis)** **^2^**

**[32] Preoperative radiotherapy**

**[33] Preoperative CT (preoperative chemotherapy)**

**[34] Preoperative corticosteroids**

**[35] Previous antibiotics (3 months)**

**[36] Yeast colonization**

**[37] Parenteral nutrition**

**[38] Previous AF (previous antifungal treatment)**

**[39] CV catheter (central venous catheter)**

**[40] SOFA (first surgery)**

**[41] SOFA (day of reoperation)**

**[42] DeltaSOFA (Difference between the SOFA score on the day of the initial surgery and the SOFA score on the day of the reoperation)**

**[43] SAPS II (day of reoperation)**

**[44] Leucocyte count the day of reoperation**

**[45] Colon perforation**

**[46] Small bowel perforation**

**[47] Anastomotic leak**

**[48] Other etiology (associated with diagnosis HC-IAI)**

[49] Bacteria in peritoneal fluid

**[50] Bacterial colonization**

**[51] Fungal colonization**

[52] GNB infection (Gram negative bacteria infection)

[53] GPC infection (Gram positive cocci infection)

[54] NFGNB infection (Non fermentative GNB infection)

[55] MDR infection

[56] Anaerobic infection

[57] Invasive candidiasis

[58] Appropriate ATB

[59] Number of antibiotics

**[60] Delay to surgery (Delay between HC-IAI diagnosis and re-intervention)**

**[61] CTscan**

**[62] Delay first surgery-reoperation**

[63] Percutaneous drainage

[64] Directed leakage

[65] Candida in BC (BC = blood culture)

[66] Candida in Peritoneal fluid

1. **Supplementary Material 5: Comparisons between clusters**

|  | **Cluster 1**  **(n=113)** | **Cluster 2**  **(n=96)** | **Cluster 3**  **(n=62)** | **P value** |
| --- | --- | --- | --- | --- |
| **Initial surgery** |  |  |  |  |
| **Age** | 65.50 (13.02) | 65.15 (15.19) | 62.68 (14.76) | 0.427 |
| **Female gender** | 53 (47) | 35 (36.5) | 31 (50.0) | 0.173 |
| **Charlson comorbidity index** | 4.30 (2.72) | 4.19 (2.54) | 4.85 (3.10) | 0.301 |
| ASA score |  |  |  | 0.019 |
| 1 | 9 (8.1) | 7 (7.3) | 2 (3.2) |  |
| 2 | 51 (45.9) | 40 (41.7) | 22 (35.5) |  |
| 3 | 51 (45.9) | 39 (40.6) | 34 (54.8) |  |
| 4 | 0 (0.0) | 10 (10.4) | 4 (6.5) |  |
| Metastasis | 27 (23.9) | 3 (3.1) | 13 (21.0) | <0.001 |
| Body mass index, kg/m^2^ | 24.05 (4.99) | 25.49 (5.95) | 22.79 (6.22) | 0.013 |
| Risk factors for invasive candidiasis ^2^ | 1.87 (1.08) | 0.32 (0.57) | 2.97 (1.19) | <0.001 |
| At least one risk factor | 102 (90.3) | 26 (27.1) | 62 (100.0) | <0.001 |
| Risk factors >2 (yeast in peritoneal fluid) ^5^ | 1 (0.9) | 39 (40.6) | 38 (61.3) | <0.001 |
| Bacterial colonization | 27 (23.9) | 4 (4.2) | 34 (56.7) | <0.001 |
| Fungal colonization | 4 (3.5) | 2 (2.1) | 15 (24.2) | <0.001 |
| Known yeast colonization (3 months) | 4 (3.5) | 1 (1.0) | 11 (17.7) | <0.001 |
| Previous antibiotics (3 months) | 13 (11.5) | 13 (13.5) | 41 (66.1) | <0.001 |
| Antibiotic as risk factor | 13 (11.5) | 45 (46.9) | 37 (59.7) | <0.001 |
| Previous antifungal treatment (3 months) | 0 (0.0) | 0 (0.0) | 9 (14.5) |  |
| Central venous catheter | 50 (44.2) | 10 (10.4) | 44 (71.0) | <0.001 |
| Malnutrition ^a^ | 27 (23.9) | 12 (12.5) | 22 (35.5) | 0.003 |
| Parenteral nutrition | 11 (9.7) | 0 (0.0) | 12 (19.4) | <0.001 |
| Hemodynamic as a risk factor | 40 (35.4) | 64 (66.7) | 45 (72.6) | <0.001 |
| Long-term steroid therapy | 1 (0.9) | 1 (1.0) | 4 (6.5) | 0.036 |
| Preoperative chemotherapy | 41 (36.3) | 3 (3.1) | 27 (43.5) | <0.001 |
| Preoperative radiotherapy | 18 (15.9) | 1 (1.0) | 10 (16.1) | <0.001 |
| Emergency procedure | 1 (0.9) | 33 (34.4) | 7 (11.3) | <0.001 |
| Cancer surgery | 110 (97.3) | 45 (46.9) | 50 (80.6) | <0.001 |
| Laparotomy | 62 (54.9) | 62 (64.6) | 48 (77.4) | 0.012 |
| Laparoscopy | 49 (43.4) | 25 (26.0) | 10 (16.1) | <0.001 |
| Robotic surgery | 11 (9.7) | 0 (0.0) | 2 (3.2) | 0.004 |
| Surgery duration>5h | 54 (47.8) | 1 (1.0) | 31 (50.0) | <0.001 |
| Supra-mesocolic surgery | 13 (11.5) | 64 (66.7) | 46 (74.2) | <0.001 |
| Pancreatic surgery | 4 (3.5) | 4 (4.2) | 13 (21.0) | <0.001 |
| Small bowel surgery | 6 (5.3) | 23 (24.0) | 15 (24.2) | <0.001 |
| Colectomy | 29 (25.7) | 38 (39.6) | 4 (6.5) | <0.001 |
| Rectal surgery | 15 (13.3) | 1 (1.0) | 0 (0.0) |  |
| Total hysterectomy | 11 (9.7) | 1 (1.0) | 2 (3.2) | 0.013 |
| Endoscopic procedure | 19 (16.8) | 5 (5.2) | 6 (9.7) | 0.027 |
| SOFA score the day of initial surgery | 0.85 (1.23) | 1.46 (2.81) | 3.15 (2.68) | <0.001 |
| **Reoperation** |  |  |  |  |
| SOFA (day of reoperation) | 3.52 (2.30) | 6.41 (3.83) | 6.40 (3.04) | <0.001 |
| Delta SOFA ^b^ | 2.69 (2.29) | 5.17 (3.92) | 3.29 (3.73) | <0.001 |
| SAPS II | 32.36 (12.79) | 50.98 (20.29) | 41.69 (14.39) | <0.001 |
| Leucocytes count (day 1) | 12.28 (8.60) | 16.89 (11.80) | 17.61 (10.75) | 0.001 |
| Gram negative bacteria infection | 71 (62.8) | 37 (38.5) | 37 (59.7) | 0.001 |
| NFGNB infection | 14 (12.4) | 11 (11.5) | 10 (16.1) | 0.678 |
| MDR infection | 18 (15.9) | 12 (12.5) | 21 (33.9) | 0.002 |
| Gram positive cocci infection | 25 (22.1) | 9 (9.4) | 31 (50) | 0.001 |
| **Antibiotic treatment** |  |  |  | 0.149 |
| Inadequate | 17 (15.0) | 12 (12.5) | 16 (25.8) |  |
| Adequate | 66 (58.4) | 57 (59.4) | 36 (58.1) |  |
| Empirical | 30 (26.5) | 27 (28.1) | 10 (16.1) |  |
| Number of antibiotics used | 2.18 (1.17) | 2.29 (0.97) | 3.44 (1.50) | <0.001 |
| **Outcomes** |  |  |  |  |
| Invasive candidiasis | 7 (6.2) | 18 (18.8) | 28 (45.2) | <0.001 |
| Candida in blood culture | 0 (0.0) | 2 (2.1) | 8 (12.9) | <0.001 |
| Candida in peritoneal fluid | 7 (6.2) | 18 (18.8) | 22 (35.5) | <0.001 |
| Percutaneous drainage (CT-scan) | 6 (5.3) | 6 (6.2) | 9 (14.5) | 0.074 |
| EAF | 12 (10.6) | 45 (46.9) | 36 (58.1) | <0.001 |
| Composite criteria | 30 (26.5) | 47 (49.0) | 35 (56.5) | <0.001 |
| 90-day mortality | 6 (5.3) | 22 (22.9) | 11 (17.7) | 0.01 |

Results are expressed as mean (standard deviation) or n (%).

ASA score, American Society of Anesthesiologists Physical Status Classification System; SOFA, Sequential Organ Failure Assessment; HC-IAI, health care-associated intra-abdominal infection; CT, computed tomography; Delta SOFA, difference between the SOFA score on the day of the initial surgery and the SOFA score on the day of the reoperation; SAPS II, Simplified Acute Physiology Score II; MDR infection, Multidrug resistant bacteria infection; NFGNB infection, non-fermentative gram negative bacterial infection; EAF, empirical antifungal treatment. a Malnutrition, body mass index < 18.5 or weight loss of over 3 kgs in 3 months. b Delta SOFA, difference between the SOFA score on the day of the initial surgery and the SOFA score on the day of the reoperation.

1. **Supplementary Material 6**

**Confusion Matrix**

|  | Cluster 1 | Cluster 2 | Cluster 3 |
| --- | --- | --- | --- |
| Cluster 1 | 32 | 1 | 1 |
| Cluster 2 | 1 | 27 | 3 |
| Cluster 3 | 0 | 0 | 14 |

The confusion matrix presents the results of classification using the linear SVM classifier in a summarized form, comparing model predictions with real classes.

1. **Supplementary Material 7**

**Statistics by Clusters**

|  | Cluster 1 | Cluster 2 | Cluster 3 |
| --- | --- | --- | --- |
| Sensitivity^a^ | 0.9697 | 0.9643 | 0.7778 |
| Specificity^b^ | 0.9565 | 0.9216 | 1.0000 |
| Positive predictive value^c^ | 0.9412 | 0.8710 | 1.0000 |
| Negative predictive value^d^ | 0.9778 | 0.9792 | 0.9385 |
| Prevalence^e^ | 0.4177 | 0.3544 | 0.2278 |
| Detection rate^f^ | 0.4051 | 0.3418 | 0.1772 |
| Detection prevalence^g^ | 0.4304 | 0.3924 | 0.1772 |
| Balanced accuracy^h^ | 0.9631 | 0.9429 | 0.8889 |

1. **Sensitivity (True Positive Rate):**
   - Sensitivity measures the proportion of true positive observations (belonging to the positive class) correctly identified by the model. Sensitivity=True Positives/True Positives + False Negatives​
2. **Specificity (True Negative Rate) :**
   - Specificity measures the proportion of true negative observations (not belonging to the positive class) correctly identified by the model. Specificity=True Negatives/True Negatives + False Positives
3. **Positive Predictive Value (Precision) :**
   - Positive Predictive Value measures the proportion of predicted positive observations that are truly positive. Positive Predictive Value=True Positives/True Positives + False Positives
4. **Negative Predictive Value :**
   - Negative Predictive Value measures the proportion of predicted negative observations that are truly negative. Negative Predictive Value=True Negatives/True Negatives + False Negatives ​
5. **Prevalence:**
   - Prevalence is the proportion of actual positive class observations in the dataset. Prevalence=True Positives + False Negatives/Total Observations
6. **Detection Rate :**
   - Detection Rate measures the proportion of observations predicted as positive by the model. Detection Rate=True Positives + False Positives/Total Observations ​
7. **Detection Prevalence:**
   - Detection Prevalence is the proportion of observations predicted as positive by the model among all observations predicted as positive. Detection Prevalence=True Positives + False Positives/True Positives + False Positives + True Negatives + False Negatives
8. **Balanced Accuracy:**
   - Balanced Accuracy is the arithmetic mean of Sensitivity and Specificity. It provides an overall measure of the model's performance. Balanced Accuracy=Sensitivity + Specificity/2

These metrics help assess different aspects of the model's performance based on the specific nature of the classification problem.

**Supplementary Fig .1 : Multivariate analysis for use of EAF**

**
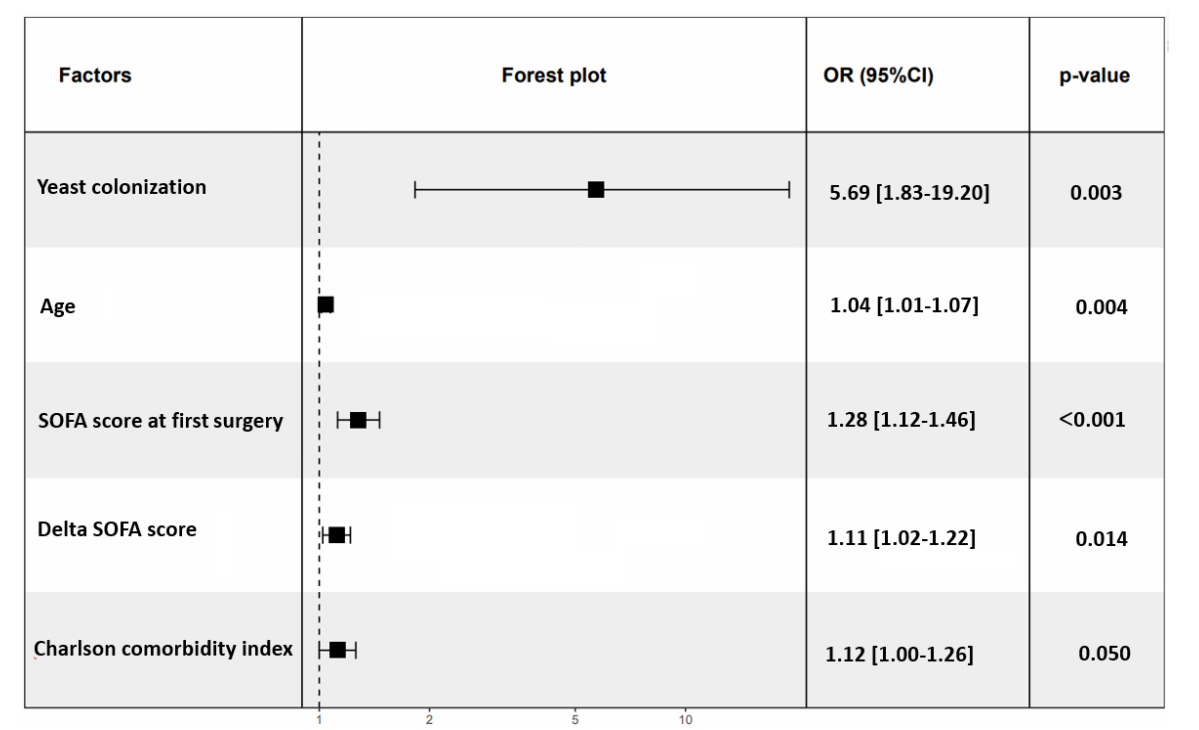
** Independent factors associated with empirical antifungal therapy (multivariate analysis). OR, odd ratio; CI, confidence interval; SOFA, Sequential Organ Failure Assessment; Delta SOFA score, Difference between the SOFA score on the day of the initial surgery and the SOFA score on the day of the reoperation.

**Supplementary Fig .2 : Standardized Mean Differences ( overlap vs unweighted cohort)**

Effect of overlap weighting on covariate balance across patients exposed or not to an early empirical antifungal therapy. SOFA, Sequential Organ Failure Assessment; Delta SOFA score, difference between the SOFA score on the day of the initial surgery and the SOFA score on the day of the reoperation; Delay to surgery, delay between health care-associated intra-abdominal infection diagnosis and re-intervention.
The absolute standardized mean difference (SMD) is the absolute value of the difference in mean between groups divided by the standard deviation. An absolute SMD less than or equal to 0.10 indicates good balance.

**Supplementary Fig.3:** **Clustering methodological design.**

The clustering methodology follows a three-step approach, integrating hierarchical clustering subsequent to dimensionality reduction through Factor Analysis of Mixed Data (FAMD). Initially, a dataset comprising 66 variables across 271 patients was selected. Utilizing FAMD, the dimensionality was effectively reduced from 66 variables to 32 meaningful dimensions. Following this, unsupervised ascendant hierarchical cluster analysis was performed by computing Euclidean distances on these dimensions. This process culminated in the formation of three well-defined clusters, employing Ward's method as the criteria for cluster merging. Ultimately, this method facilitates the grouping of patients based on shared clinical and biological profiles.

**Supplementary Fig.4**

The presented scree plot illustrates the proportions of total data variance explained by different axes (or dimensions) extracted during factorial analysis. Each axis is a linear combination of the initial variables. The variances of the 66 dimensions exhibit a gradual decrease, with the top 32 dimensions collectively explaining over 80% of the total variance. Focusing solely on these 32 dimensions captures a substantial portion of the information from all 66 dimensions. This suggests the possibility of reducing dimensionality by focusing on the most significant dimensions.

**Supplementary Fig.5**

**
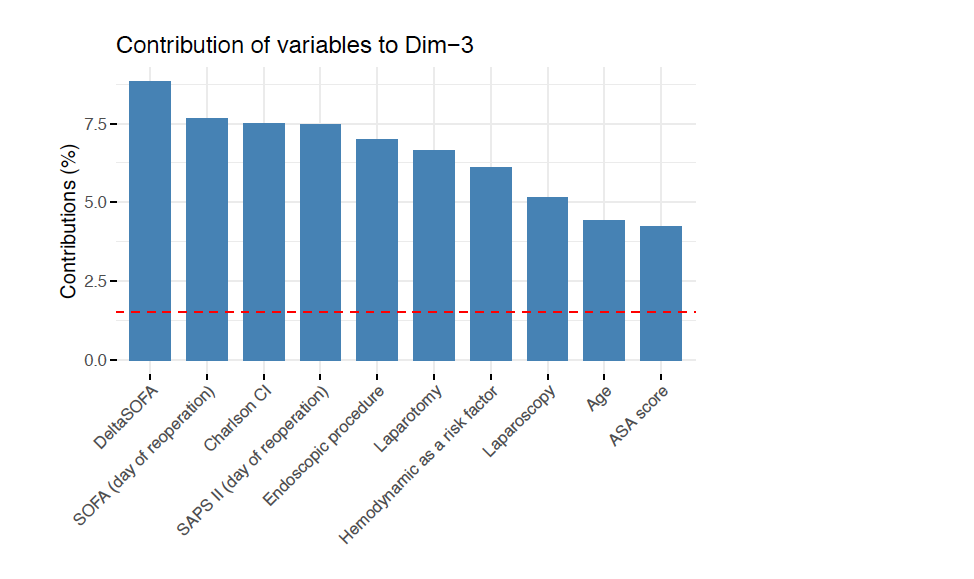
** **
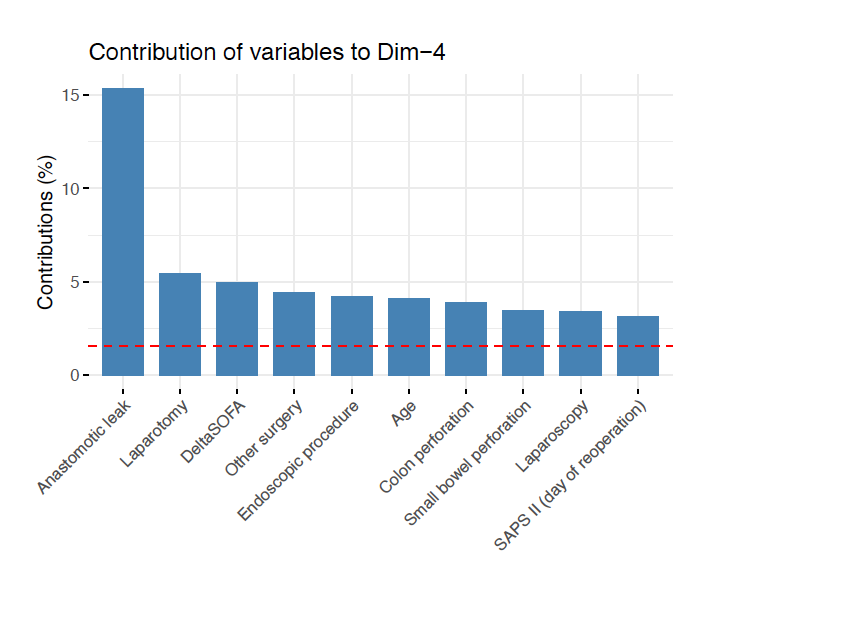
**


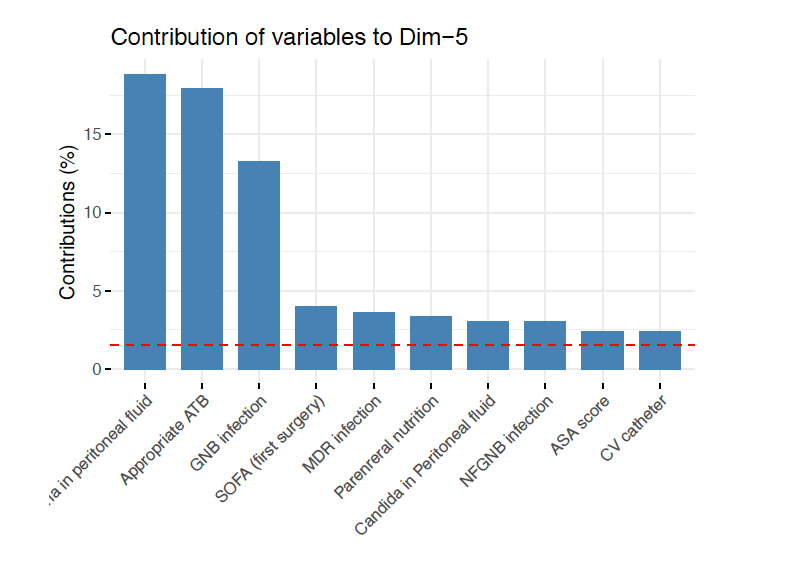


Contribution of clinical and biological variables to the first five dimensions. Factor analysis generates dimensions, which are linear combinations of the original variables. Each dimension represents an underlying structure in the data, and the contribution of clinical and biological variables to these dimensions can be interpreted to understand which features or aspects of the data are primarily captured by each dimension. This graph illustrates how clinical variables (such as patient-related characteristics) and biological variables contribute to the formation of the first five dimensions.

**Supplementary Fig.6: Accuracy of the SVM classifier**

The plot is showing that the SVM classifier is giving best accuracy (0.876) on C = 0.01. This means that the vertical axis of the graph represents model accuracy, which is a measure of the quality of the model's predictions. An accuracy of 0.876 means that the model is correct around 87.6% of the time when making predictions on new data. The SVM model depends on a parameter called "C", which controls the penalty for misclassification. A value of C=0.01 indicates that the C parameter used in the SVM model is set to 0.01. This suggests that, for these specific conditions, a C parameter equal to 0.01 gives the best results in terms of model accuracy.

**References**

1. Dupont H, Bourichon A, Paugam-Burtz C, Mantz J, Desmonts JM. Can yeast isolation in peritoneal fluid be predicted in intensive care unit patients with peritonitis?*: *Crit Care Med*. 2003;31(3):752-757. doi:10.1097/01.CCM.0000053525.49267.77

2. Thomas-Rüddel DO, Schlattmann P, Pletz M, Kurzai O, Bloos F. Risk Factors for Invasive Candida Infection in Critically Ill Patients: A Systematic Review and Meta-analysis. *Chest*. 2022;161(2):345-355. doi:10.1016/j.chest.2021.08.081

3. Lê S, Josse J, Husson F. FactoMineR: An R Package for Multivariate Analysis. *J Stat Softw*. 2008;25:1-18. doi:10.18637/jss.v025.i01

4. Husson F, Josse J. Multivariate Data Analysis - Special focus on Clustering and Multiway Methods.

5. Dupont H. Predictive Factors of Mortality Due to Polymicrobial Peritonitis With Candida Isolation in Peritoneal Fluid in Critically Ill Patients. *Arch Surg*. 2002;137(12):1341. doi:10.1001/archsurg.137.12.1341
